# Setting positive end-expiratory pressure: lung and diaphragm ultrasound

**DOI:** 10.1097/MCC.0000000000001119

**Published:** 2023-11-17

**Authors:** Amne Mousa, Peter Klompmaker, Pieter R. Tuinman

**Affiliations:** a Department of Intensive Care, Amsterdam UMC location Vrije Universiteit Amsterdam; b Amsterdam Cardiovascular Sciences research institute, Amsterdam UMC; c Amsterdam Leiden Intensive Care Focused Echography (ALIFE), Amsterdam, The Netherlands

**Keywords:** diaphragm, lung, mechanical ventilation, positive end-expiratory pressure, ultrasonography

## Abstract

**Purpose of review:**

The purpose of this review is to summarize the role of lung ultrasound and diaphragm ultrasound in guiding ventilator settings with an emphasis on positive end-expiratory pressure (PEEP). Recent advances for using ultrasound to assess the effects of PEEP on the lungs and diaphragm are discussed.

**Recent findings:**

Lung ultrasound can accurately diagnose the cause of acute respiratory failure, including acute respiratory distress syndrome and can identify focal and nonfocal lung morphology in these patients. This is essential in determining optimal ventilator strategy and PEEP level. Assessment of the effect of PEEP on lung recruitment using lung ultrasound is promising, especially in the perioperative setting. Diaphragm ultrasound can monitor the effects of PEEP on the diaphragm, but this needs further validation. In patients with an acute exacerbation of chronic obstructive pulmonary disease, diaphragm ultrasound can be used to predict noninvasive ventilation failure. Lung and diaphragm ultrasound can be used to predict weaning outcome and accurately diagnose the cause of weaning failure.

**Summary:**

Lung and diaphragm ultrasound are useful for diagnosing the cause of respiratory failure and subsequently setting the ventilator including PEEP. Effects of PEEP on lung and diaphragm can be monitored using ultrasound.

## INTRODUCTION

The choice of ventilator settings depends upon the underlying cause of respiratory failure and condition of the patient [1]. Positive end-expiratory pressure (PEEP) is one of the cornerstones of any ventilation strategy but remains one of the most difficult parameters to set; both too low or too high PEEP can have detrimental effects. A balance must be reached between lung recruitment and improved gas exchange versus overdistention and hemodynamic consequences [1]. Several bedside tools, such as plateau pressures, PEEP/FiO2 tables and oesophageal manometry, are used to set PEEP. However, thus far, none of these methods has improved outcomes in clinical trials [1].

In addition, careful bedside assessment is needed to evaluate both the positive and negative effects of PEEP. Critical care ultrasonography, increasingly used in daily practice, is a promising tool for bedside assessment in this matter [2]. Both lung ultrasound and diaphragm ultrasound are frequently used for diagnosing and monitoring patients with acute respiratory failure [3,4^▪^,5^▪^] and their use has a major impact on patient management, including ventilator settings [6].

In this narrative review, we discuss the role of lung ultrasound and diaphragm ultrasound in personalisation of ventilator settings. We give a brief overview of how lung ultrasound and diaphragm ultrasound is used to determine underlying causes of respiratory failure and how to set the ventilator with an emphasis on PEEP. Furthermore, we will elaborate on how to use these ultrasound modalities during the weaning phase. Recent advances for using lung ultrasound and diaphragm ultrasound to assess the effects of PEEP will be discussed. 

**Box 1:**
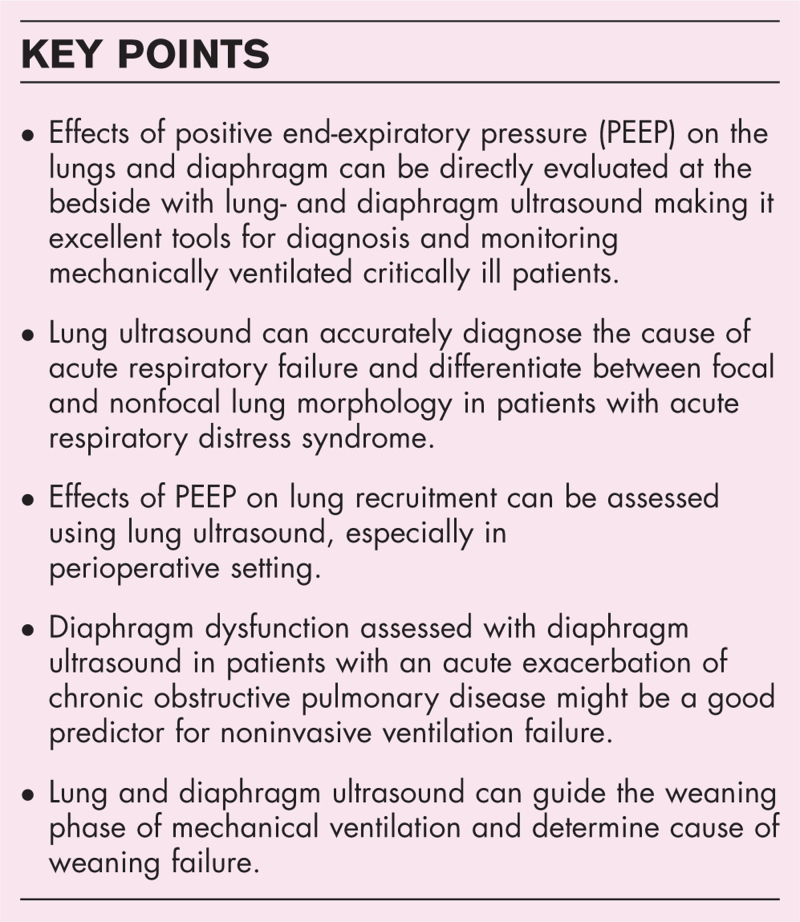
no caption available

## EFFECTS OF POSITIVE END-EXPIRATORY PRESSURE ON LUNGS AND DIAPHRAGM

**FIGURE 1:**
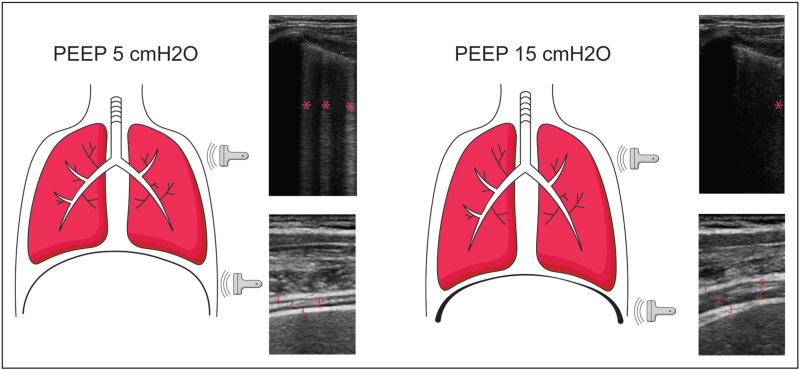
Effects of low and high PEEP on the lungs and diaphragm. PEEP increases expiratory lung volume, thereby changing the fluid to gas ratio, resulting in change of detected lung ultrasound pattern (B-profile to A-profile). In addition, PEEP causes the diaphragm to be shortened, resulting in a thicker end-expiratory muscle with potentially less efficient contractility. ^∗^indicates individual B-lines. 1: pleura, 2: diaphragm muscle, 3: peritoneum. | shows thickness measurement of the diaphragm muscle. PEEP, positive end-expiratory pressure.

Lung ultrasound is able to accurately detect and monitor acute pulmonary pathologies [7,8]. Lung ultrasound-patterns (A-, B- and C-patterns) are dependent on the fluid-to-gas ratio of the pulmonary parenchyma [9]. For details about image acquisition and interpretation, we refer to a recent review [10^▪^]. The Bedside Lung Ultrasound in Emergency (BLUE) protocol is frequently used for diagnosing pulmonary pathology in critically ill patients. This protocol, using a decision tree based on specific lung ultrasound findings, provides a diagnosis in patients with acute respiratory failure with an accuracy of around 90% [11]. To monitor effects of treatment on lung aeration, the lung aeration score or lung ultrasound score, a semiquantitative score, can be used [8]. A greater loss of aeration results in a higher lung ultrasound score: ranging from 0 to 36. Lung ultrasound is able to detect effects of PEEP on the lung, since PEEP increases end-expiratory lung volume and therefore changes the fluid to gas ratio, resulting in change of lung ultrasound patterns and/or score (Fig. 1) [12].

Diaphragm ultrasound is an excellent bedside tool for evaluating diaphragm anatomy and function [3,4^▪^]. For details about image acquisition and interpretation, we refer to a recent review and expert consensus [3,4^▪^]. Assessment of the diaphragm includes measurement of muscle thickness, contractility (thickening fraction, TF_di_) and diaphragm excursion [4^▪^]. Muscle thickness <1.5 mm indicates atrophy [3,13]. Muscle thickness is highly dependent on location of measurement, gender and patient position [3,14]. A TF_di_ in ICU patients of <30–34% is considered abnormal [3]. Diaphragm excursion <2 cm during quiet breathing without assistance of mechanical ventilation is indicative of diaphragm dysfunction [4^▪^].

It was recently found that PEEP has a direct effect on diaphragm geometry; it shortens the muscle creating an overlap of thick and thin filaments, resulting in a less optimal length for muscle contraction. PEEP can thus reduce the contractile efficiency of the diaphragm [15^▪^]. Moreover, prolonged exposure to PEEP can result in remodelling of the diaphragm. Withdrawal after prolonged exposure can result in reduced contractile efficiency by stretching the remodelled muscle fibres in the diaphragm [15^▪^]. These effects can be visualized using ultrasound; when PEEP is increased, both TF_di_ and excursion decrease whilst the thickness of the diaphragm increases (Fig. 1) [16^▪▪^].

## SETTING THE VENTILATOR USING LUNG AND DIAPHRAGM ULTRASOUND

**FIGURE 2:**
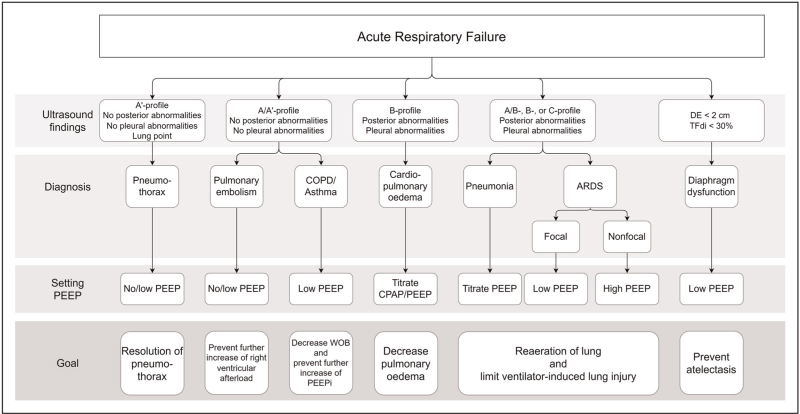
Lung or diaphragm ultrasound findings for common causes of respiratory failure and corresponding guidelines for setting PEEP. ARDS, acute respiratory distress syndrome; COPD, chronic obstructive pulmonary disease; CPAP, continuous positive airway pressure; DE, diaphragm excursion; PEEP, positive end-expiratory pressure; PEEPi, intrinsic positive end-expiratory pressure; TFdi, diaphragm thickening fraction.

The most common underlying causes of acute respiratory failure diagnosed with either lung- or diaphragm ultrasound and subsequent choice of initial ventilator settings, with emphasis on PEEP, are summarized in Fig. 2[17]. Ultrasound findings should always be used in concert with other findings and clinical features, as some ultrasound signs can be present in more than one pulmonary pathology and critically ill patients often have multiple causes for respiratory failure [5^▪^].

### Pneumothorax

Patients with pneumothorax show A-profile without lung sliding, called A’-profile, on lung ultrasound. In combination with lung point, lung ultrasound confirms pneumothorax with a specificity of 100% [10^▪^]. Positive pressures including PEEP should be kept as low as possible. Resolution of the pneumothorax can be monitored using lung ultrasound [18].

### Pulmonary embolism

Patients with pulmonary embolism present with A-profile. In some cases a shred sign is seen following a pulmonary infarction. For diagnosis of pulmonary embolism, lung ultrasound should be combined with venous ultrasound to detect deep-venous thrombosis.

In pulmonary embolism, PEEP is set as low as possible to prevent a further increase of right ventricular afterload. Cardiac ultrasound can be used to monitor effects of treatment on right ventricular function [19].

### Chronic obstructive pulmonary disease and severe asthma

Patients with exacerbation of chronic obstructive pulmonary disease (COPD) or severe asthma typically present with an A-profile on lung ultrasound since the lung parenchyma is usually not affected [10^▪^].

Noninvasive ventilation (NIV) is the first choice of respiratory support for patients presenting with an exacerbation of COPD. In assisted modes of ventilation, moderate external PEEP is applied to counterbalance intrinsic PEEP and hence to reduce effort needed to trigger the ventilator and to promote patient-ventilator synchrony [20]. In controlled modes of ventilation, low or sometimes even zero PEEP is advised to not further increase intrinsic PEEP and prevent dynamic hyperinflation.

Severe diaphragm dysfunction within the first 48 h of NIV, defined as TF_di_ <20%, is associated with an increased risk of NIV failure and worse outcomes. Diaphragm dysfunction in these patients is probably caused by dynamic hyperinflation. This results in less optimal diaphragm geometry causing the diaphragm to work less efficient. Improved diaphragm function under NIV is probably an indication of decreased hyperinflation [21]. Two prospective studies have shown that diaphragm dysfunction, especially lower excursion, identified with ultrasound can reliably predict NIV failure in emergency department settings [22^▪^,^23^]. Assessment of diaphragm function to predict NIV failure in patients without COPD exacerbation or in an ICU setting, although promising, needs further validation [24,25].

Severe asthma patients are at risk of barotrauma due to the increased flow limitations. In these patients, PEEP settings are set as low as possible to prevent further hyperinflation of the lung [20].

### Cardiogenic pulmonary oedema

Patients with pulmonary congestion present with multiple, diffuse bilateral B-lines (>2 per view, so called B-profile) and regular thin pleura on lung ultrasound [26^▪^,^27^]. Applying PEEP through both noninvasive and invasive ventilation can be of great benefit in the treatment of cardiogenic pulmonary oedema (CPE) [28].

Lung ultrasound can be used to evaluate the effect of PEEP by monitoring the number of- and regions with B-lines. In the prehospital emergency setting, lung ultrasound was found to be a reliable tool for monitoring effectiveness of treatment with PEEP [29,30]. Although no studies investigated lung ultrasound-guided PEEP levels, guidelines recommend monitoring treatment effect with bedside lung ultrasound in the clinical setting [31]. The consequences of too high PEEP – e.g. hemodynamic compromise, should be monitored.

### Pneumonia and Acute respiratory distress syndrome

Patients with pneumonia can present with unilateral B-profile (A/B-profile), bilateral B-profile, C-profile and/or posterior consolidations. Moreover, presence of dynamic air bronchograms and colour Doppler flow in consolidations are highly suggestive of pneumonia [32].

Differentiation between CPE and noncardiogenic oedema can be challenging. Nonetheless, recent studies show that lung ultrasound can accurately differentiate between these two diagnoses [26^▪^,33^▪^]. Acute respiratory distress syndrome (ARDS), the most frequent cause of noncardiogenic oedema in ICU patients, is characterized by nonhomogenous distribution of B-lines and/or pleural abnormalities. In patients with pneumonia and/or ARDS, ventilator settings should be set carefully to maintain sufficient gas exchange but limit ventilator-induced lung injury by using low tidal volumes, driving pressure and titration of PEEP [1]. The use of ultrasound to titrate PEEP in patients with pneumonia or ARDS is discussed in the paragraph ‘assessment of lung aeration’.

### Diaphragm dysfunction

A less common cause of acute respiratory failure is diaphragm dysfunction. Diaphragm ultrasound can diagnose both weakness and paralysis of the (hemi-)diaphragm. In these patients, PEEP is set to prevent further atelectasis. Although, titration of pressure support for adequate ventilation is more important.

In recent years, diaphragm-protective ventilation in addition to lung-protective ventilation has become an important field of research [34^▪^,^35^]. It has been proposed that both over- and under assistance of the diaphragm should be prevented. As potential target range, based on physiological data, a TF_di_ between 15% and 30–40% should be maintained [13]. A recent study found that titration of inspiratory support based on patient breathing effort greatly increased the time that patients had diaphragm effort in the predefined diaphragm-protective range without compromising tidal volumes and transpulmonary pressures [34^▪^]. However, in this study transdiaphragmatic pressure measurements were used for assessment of diaphragm effort.

## ASSESSMENT OF LUNG AERATION

### Acute respiratory distress syndrome lung morphology

**FIGURE 3:**
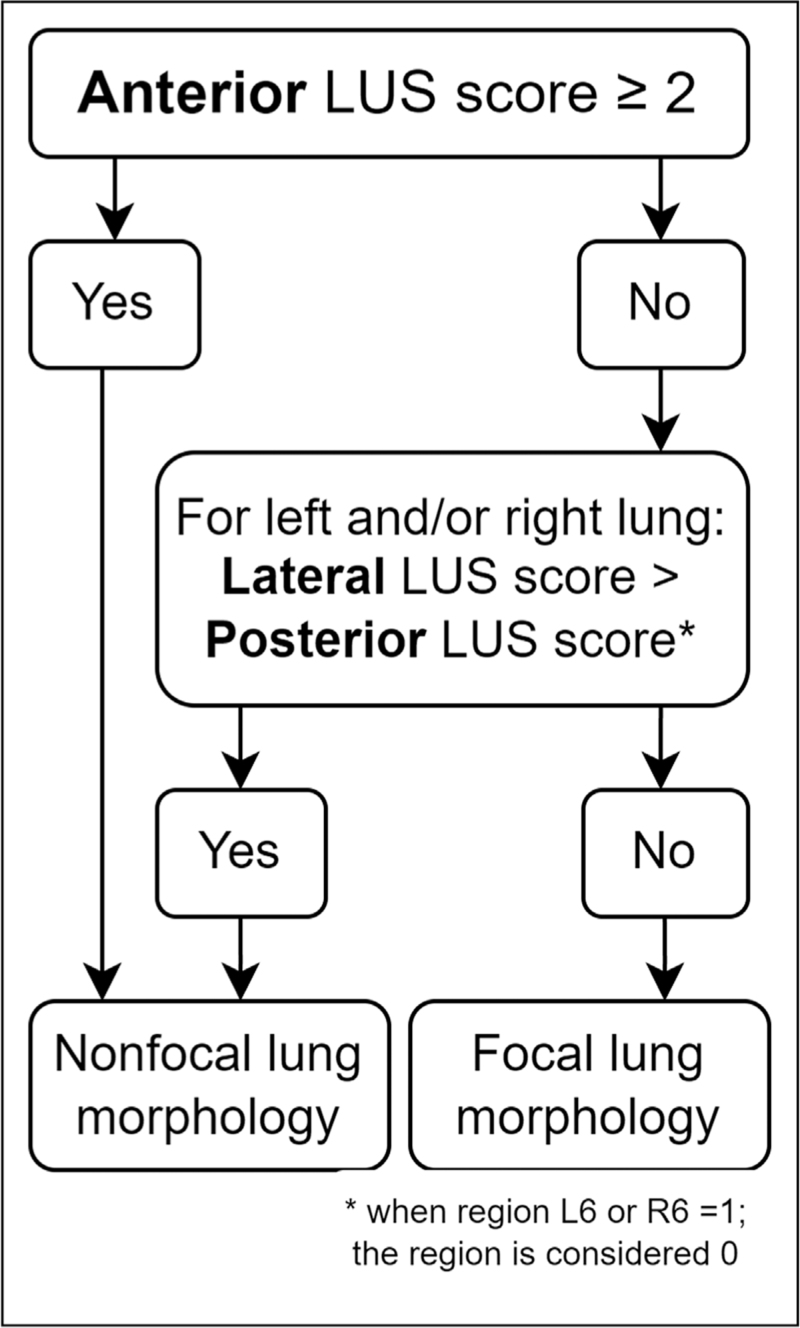
The Amsterdam method for lung ultrasound assessment to differentiate between focal and nonfocal lung morphology in patients with acute respiratory distress syndrome. Adapted from Pierrakos *et al.* (2021).

Two distinct ARDS phenotypes, focal and nonfocal, have been identified [36]. Focal ARDS is considered to respond to low PEEP and prone positioning whereas nonfocal ARDS, with patchy loss of aeration, generally responds to high PEEP and recruitment manoeuvres [36]. The largest study to date where individualisation of ventilator strategy based on these phenotypes was researched mostly used chest radiography for identification of the phenotypes with a great degree of misclassification [37]. A reduction in mortality was found after correcting for misclassification. Lung ultrasound can accurately identify these phenotypes with the Amsterdam method being the advised method (Fig. 3) [38^▪▪^]. Using lung ultrasound to identify these phenotypes and guide ventilator strategy accordingly is currently being investigated (ClinicalTrials.gov; identifier NCT05492344).

### Ultrasound-guided recruitment

**FIGURE 4:**
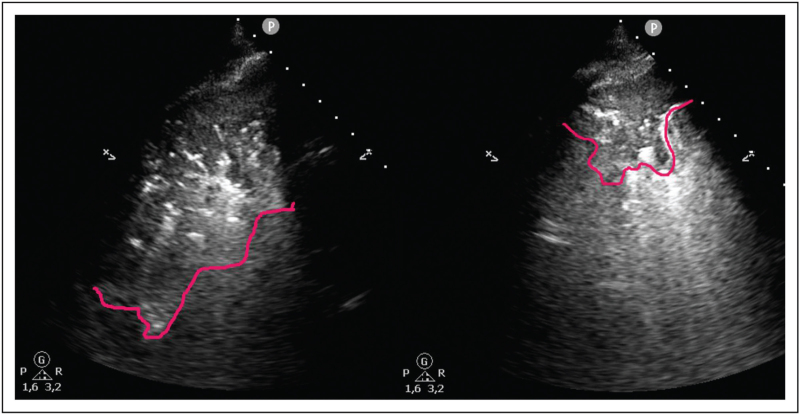
Effect of PEEP on reaeration of the lung. Increase of PEEP can result in recruitment of lung tissue which can be seen on lung ultrasound as a smaller consolidation when PEEP is increased from PEEP 5 (left) to PEEP 15 (right). The lines indicate the border of the consolidations. PEEP, positive end-expiratory pressure.

In patients with pneumonia and/or ARDS, PEEP is mostly used to preserve lung aeration. To set PEEP in these patients recruitability should be assessed to help identify patients who are likely to benefit from higher PEEP levels (Fig. 4). Studies researching the effects of PEEP on lung recruitment assessed by lung ultrasound have found conflicting results [39,40,41^▪▪^]. While one study found that lung ultrasound was able to accurately assess reaeration and identify differences in lung ultrasound scores at different PEEP levels [41^▪▪^], other studies found that lung ultrasound was unable to consistently detect these changes when PEEP levels were changed [39,40]. These different outcomes might be explained by the fact that changes in consolidation size or a decrease in the number of B-lines are not taken into account. Thus smaller PEEP-induced aeration changes are not always detected. A small pilot study found that by taking these smaller changes into account it was possible to accurately assess aeration changes [42].

An important limitation of lung ultrasound is its inability to directly asses overdistention. However, there are some promising experimental studies evaluating the potential of lung ultrasound for detecting overdistension such as quantification of lung sliding [43,44] and ultrasound elastography [45].

Although diaphragm ultrasound is not commonly used for assessing lung aeration, a small study in patients with ARDS investigated diaphragm ultrasound for assessing aeration [46]. They found an increase in diaphragm excursion in the dorsal region after an increase of PEEP. This suggests an increase in lung volume due to increased aeration after recruitment manoeuvres. Interestingly, they did not find a relation between increase in PEEP and diaphragm excursion in ventral regions. Since overdistension of the lungs is usually present in the ventral regions during mechanical ventilation, one can hypothesize that a lack of excursion in the ventral regions might be suggestive of overdistension. This needs further studies.

In the perioperative setting, the use of lung ultrasound to set PEEP has been extensively studied. The majority of patients undergoing general anaesthesia develop atelectasis, which is associated with worse postoperative outcomes. Therefore, decreasing loss of aeration seems a logical target for improving postoperative outcomes [47]. Lung ultrasound accurately detects reaeration of lung tissue after application of PEEP during surgery [47]. Lung ultrasound-guided recruitment and PEEP titration – i.e. recruitment manoeuvres when lung ultrasound detected atelectasis at prespecified time points - improved aeration and oxygenation intraoperatively, but did not result in reduced pulmonary complications [48^▪▪^,^49^].

## THE ROLE OF LUNG AND DIAPHRAGM ULTRASOUND DURING WEANING

Identifying patients who are ready to be weaned of mechanical ventilation is often challenging. Both delayed weaning and weaning failure are associated with high mortality. Therefore correct identification of patients ready to be weaned is vital [50]. Lung and diaphragm ultrasound is useful in in this context.

### Role of lung ultrasound

A decrease or removal of PEEP can result in derecruitment which can be detected with lung ultrasound. After a spontaneous breathing trial (SBT), a lung ultrasound score of <13 is highly predictive of extubation success with a negative likelihood ratio of 0.20, whereas a score of >17 is highly predictive of failure with a positive likelihood ratio of 11.8 [51].

A decrease in intrathoracic pressure during weaning due to decreased PEEP can induce weaning induced pulmonary oedema (WIPO). This can be detected by lung ultrasound as development of B-lines. Patients with an increase of ≥6 B-lines, in four anterior regions, during a SBT showed a high risk of SBT failure with a sensitivity of 88% and specificity of 91% [52].

### Role of diaphragm ultrasound

Diaphragm dysfunction is an important complication of mechanical ventilation and is associated with poor clinical outcomes, including weaning failure [13]. Diaphragm excursion of >1–1.5 cm (sensitivity 85%, specificity 75%) and a TF_di_ of 30–36% (sensitivity 80%, specificity 80%) are predictive of successful extubation [3,53^▪^,^54^]. In patients after a successful SBT, diaphragm dysfunction detected by ultrasound, was not associated with an increased risk of weaning failure [55]. This indicates that a single ultrasound measurement should not be used to base clinical decisions on.

### Holistic approach

Weaning is a stress test of the whole cardiorespiratory system. A more holistic ultrasound assessment of the heart, lung and diaphragm might therefore be more suitable to assess if the patient is ready to wean [3]. Thoracic ultrasound assessment, including heart, lung and diaphragm ultrasound, especially the presence of pulmonary oedema and increased left ventricle pressures are good measures (positive likelihood ratio of 22.9 and negative likelihood ratio of 0.16 when ultrasound examination is performed prior to a SBT) for predicting weaning failure [56]. Furthermore, this assessment was able to identify the cause of weaning failure in all patients [56]. However, in patients who successfully passed a SBT, thoracic ultrasound seemed to have less relevance for prediction of respiratory distress after extubation [57]. In summary, it is advised to use an ultrasound assessment of all components of this cardiorespiratory system to detect the cause of weaning failure and guide treatment [3].

## CONCLUSION

Lung ultrasound and diaphragm ultrasound are excellent tools for diagnosing the cause of acute respiratory failure, including lung morphology in ARDS patients. Thereby it can guide and monitor ventilator settings including PEEP. Ultrasound is able to detect PEEP induced changes on the lungs and diaphragm. Using lung ultrasound to assess recruitability and set PEEP in critically ill patients needs further validation, but is useful perioperatively. In patients with acute exacerbation of COPD, diaphragm ultrasound allows for prediction of NIV failure. During weaning of mechanical ventilation combined ultrasound of lung, diaphragm and heart could potentially be used to help predict weaning failure and to determine the cause of weaning failure.

## Acknowledgements

*We thank M. Otten for his contribution to the figures in this article.*

### Financial support and sponsorship

*None.*

### Conflicts of interest

*There are no conflicts of interest.*

## REFERENCES AND RECOMMENDED READING

Papers of particular interest, published within the annual period of review, have been highlighted as:

▪ of special interest
▪▪ of outstanding interest
